# STEM CELLS HOMEOSTASIS AND AGING

**DOI:** 10.1093/geroni/igad104.0862

**Published:** 2023-12-21

**Authors:** Leanne Jones, Emmanuelle Passegue

## Abstract

Adult (tissue) stem cells support tissue homeostasis and repair throughout the life of an individual. Numerous changes occur with age that result in altered stem cell behavior and reduced tissue maintenance and regeneration. Changes can be cell autonomous including changes in cell cycle progression, decreased bioenergetic efficiency, increased DNA damage, and epigenetic alterations. In addition, poorly understood changes to the local and systemic environments occur that result in decreased stem cell activity or alterations in commitment or differentiation potential. This symposium will cover the impact of aging on multiple adult stem cell systems, including hematopoietic stem cells (HSCs), intestinal stem cells (ISCs), and neural stem cells.

